# Etiology of serum vitamin B_12_ elevation 1 month after bariatric surgery: A case-control study based on China population

**DOI:** 10.1097/MD.0000000000028071

**Published:** 2021-12-23

**Authors:** Pengsen Guo, Huawu Yang, Jinhua Zhou, Rui Mao, Dafang Zhan, Tongtong Zhang, Jiang Yuan, Yanxi Ou, Yanjun Liu

**Affiliations:** a College of Medicine, Southwest Jiaotong University, The Third People's Hospital of Chengdu, Chengdu, Chengdu, Sichuan Province, China; b The Center of Gastrointestinal and Minimally Invasive Surgery, The Third People's Hospital of Chengdu, Chengdu, Sichuan Province, China; c Medical Research Center, The Third People's Hospital of Chengdu, The Second Affiliated Hospital of Chengdu, Chongqing Medical University, Chengdu, Sichuan Province, China; d Medical Service Center of Sichuan Province, Chengdu, Sichuan Province, China; e Qiqihar Medical University, Qiqihar, Heilongjiang Province, China.

**Keywords:** bariatric surgery, case-control, obesity, vitamin B_12_

## Abstract

Few studies have reported an increase in vitamin B_12_ (VitB_12_) levels after bariatric surgery. This study reports the phenomenon and adverse reactions of serum VitB_12_ elevation 1 month after surgery and explores the possible etiologies.

Retrospective analysis was performed on VitB_12_ data for 112 patients from January 2018 to October 2019. Then, 87 patients were included between November 2019 and August 2020. They were divided into 2 groups according to the level of VitB_12_ after surgery, and the demographic and clinical data were analyzed. Then, LASSO regression model analysis and multiple logistic regression analysis were performed to explore the risk factors for VitB_12_ elevation after surgery.

Retrospective data showed that the VitB_12_ level was significantly increased 1 month after surgery. Comparison of data between the 2 groups found that more patients also had diabetes in the nonelevated group. The postoperative folic acid and VitB_12_ levels of the elevated group were significantly higher than those of the nonelevated group. More patients had concurrent constipation in the elevated group than in the nonelevated group. Two meaningful variables in LASSO regression analysis were incorporated into the multivariate logistic regression analysis, and constipation was found to be an independent risk factor for the increase in VitB_12_ after surgery. Of the 199 patients in this study, 111 patients had elevated VitB_12_ levels after surgery. Among them, 7 patients had peripheral nerve symptoms.

Constipation is an independent risk factor for increased VitB_12_ levels after surgery. High levels of VitB_12_ may cause some peripheral nerve symptoms. Therefore, it is necessary to pay attention to patients with postoperative constipation, monitor their VitB_12_ level as soon as possible, and take measures to improve constipation to avoid some adverse reactions caused by elevated VitB_12_ levels.

## Abstract 2

Supplemental Digital Content is available in the text

## 1 Introduction

Obesity is a complex multifactorial disease. The worldwide prevalence of overweight and obesity has doubled since 1980 to the extent that nearly one-third of the world's population is now classified as overweight or obese.^[1]^ Obesity is caused by improper fat generation and is a metabolic disease that seriously endangers health.^[2]^ It increases the risk for developing multiple diseases, such as hypertension, diabetes, cardiovascular disease,^[3]^ several types of cancers,^[4]^ and poor mental health.^[5]^ All of these affect quality of life and work efficiency and increase medical costs. It has been proven that weight loss can effectively prolong life and reduce medical economic burden.^[6]^ In the treatment of obesity, physical therapy, such as moderate exercise, diet adjustment, and oral or injection drugs are inefficient, making it easy to regain weight, while surgery can achieve sustained and effective weight loss and is considered to be the most effective and lasting method for the treatment of obesity.^[7]^

Currently, the most commonly used method of bariatric surgery is laparoscopic sleeve gastrectomy (LSG) and laparoscopic Roux-en-Y gastric bypass (RYGB).^[8]^ Besides, in China, more and more LSG + jejunal bypass (JJB) used to treat patients who are heavier. LSG + JJB is on the basis of Sleeve, idling a section of the small intestine further reduces absorption to achieve better weight loss. Clinical studies have shown that although bariatric surgery can effectively reduce the weight of obese patients, postoperative malnutrition caused by bariatric surgery deserves our attention, and vitamin B_12_ (VitB_12_) is the most commonly affected nutrient.^[1,9,10]^ VitB_12_ is mainly absorbed at the end of the ileum and requires internal factors secreted by gastric parietal cells.^[11]^ The amount of VitB_12_ stored in the body is approximately 2000 μg, and only 2 μg is needed on average every day. Despite such large reserves, VitB_12_ deficiency is common after bariatric surgery.^[12]^ Malabsorption and inadequate oral intake are the 2 main causes of VitB_12_ deficiency in patients undergoing bariatric surgery.^[11]^ Other causes of VitB_12_ deficiency include postoperative food intolerance and bacterial overgrowth syndrome.^[13]^

However, there are few reports of elevated VitB_12_ levels after bariatric surgery. High serum levels of VitB_12_ after surgery may lead to some peripheral nerve symptoms, such as limb numbness, fatigue, pain, facial edema, and whole body stiffness. In addition, 1 study has reported that high serum levels of VitB_12_ can increase the incidence of unstable angina pectoris.^[14]^ Therefore, increased serum VitB_12_ levels after bariatric surgery need our attention, as this condition may affect the prognosis of patients. The purpose of this paper is to analyze the related risk factors of an increase in serum VitB_12_ after bariatric surgery through a case-control study and to obtain the theoretical basis for the increase in serum VitB_12_ levels after bariatric surgery, providing some ideas for the prevention of postoperative VitB_12_ imbalance.

## 2 Materials and methods

Retrospective analysis was performed for serum VitB_12_ data before and at 1, 3, and 6 months after surgery for 112 obese patients (45 males and 67 females) who underwent bariatric surgery from January 2018 to October 2019 at Third People's Hospital of Chengdu. Later, the analysis included 87 obese patients (29 males and 58 females) who underwent bariatric surgery between November 2019 and August 2020 at Third People's Hospital of Chengdu. A total of 53 patients with elevated serum VitB_12_ levels (>771 pg/mL) were divided into the case group, and a total of 34 patients with nonelevated serum VitB_12_ levels (≤771 pg/mL) 1 month after surgery were divided into the control group. The general medical data of all patients were collected and recorded, including sex, age, body mass index (BMI), mode of surgery, complications (hypertension, diabetes, etc), hematological indexes (such as serum VitB_12_ level, folic acid, hemoglobin, blood lipids, uric acid, and liver and kidney function). All patients completed the surgery successfully, and a clear liquid diet was required within 1 month after surgery, with 60 g protein powder and 2 multivitamin tablets supplemented daily. The clinical data of BMI, serum VitB_12_ and folic acid were reviewed and recorded 1 month after surgery. Patients were asked if they took multivitamin tablets as prescribed by the doctor and whether there was constipation and/or acid regurgitation. The Ethics Review Committee of Chengdu Third People's Hospital approved the study. All the subjects provided and signed a written informed consent. This study has benn registered in Chinese Trial Registry. Clinical trial registration numbers and date: ChiCTR2000038232, on September 15, 2020. All methods were performed in accordance with the regulations.

Inclusion criteria included: BMI ≥ 32.5 kg/m^2^ or BMI ≥ 27.5 kg/m^2^ with high-risk comorbid conditions, such as cardiopulmonary problems (eg, severe sleep apnea and Pickwickian syndrome), uncontrolled type 2 diabetes, or obesity-induced physical problems interfering with lifestyle, hyperuricemia, renal insufficiency, or a history of primary hyperparathyroidism; patients undergoing bariatric surgery in our hospital; patients with normal serum VitB_12_ levels before surgery; and patients who understood and volunteered for the study. Exclusion criteria included: secondary obesity caused by endocrine disorders; history of malignant tumor; severe liver disease or renal failure; preoperative use of corticosteroids and other drugs; patients who needed to take antiepileptic drugs, metformin, histamine H2 receptor inhibitors and other drugs that affect VitB_12_ levels after surgery; patients deemed to have uncontrollable mental illness; patients whose comprehension and cognitive abilities were not adequate enough to complete the follow-up; other reasons affecting the metabolism of VitB_12_; and patients who failed to follow-up after surgery. The demographic and clinical data of 87 patients are presented in Table 1.

**Table 1:** Demographic and clinical data of patients.

Factors	
Age (yr)	31.29 ± 9.49
Range	14–74
Gender (n)	
Male	29
Female	58
Height (cm)	166.71 ± 8.61
Weight (kg)	105.90 ± 24.46
BMI (kg/m^2^)	37.76 ± 6.18
Mode of surgery (n)	
LSG	49
LSG + JJB	30
LRYGB	8
Hematological indexes	
Hemoglobin (g/L)	142.02 ± 14.45
Folic acid (ng/mL)	7.81 ± 3.61
Vitamin B_12_ (pg/mL)	525.42 ± 163.33
ALT (U/L)	52.19 ± 36.10
AST (U/L)	32.43 ± 17.47
Creatinine (μmol/L)	59.96 ± 13.34
Uric acid (μmol/L)	428.25 ± 124.93
Triglyceride (mmol/L)	2.04 ± 1.05
Cholesterol (mmol/L)	4.96 ± 0.85
FBG (mmol/L)	6.39 ± 3.01
HBA1c (%)	6.10 ± 1.52
Preoperative complication (n)	
T2DM	19
HBp	19
Hyperuricemia	41
Hyperlipidemia	44
SAS	56
Fatty liver	79

The results are expressed as mean ± SD or percentages. ALT = glutamic pyruvic transaminase, AST = glutamic oxaloacetic transaminase, BMI = body mass index, FBG = fasting blood glucose, HBA1c = glycosylated hemoglobin, HBp = high blood pressure, JJB = jejunal bypass, LRYGB = laparoscopic Roux-en-Y gastric bypass, LSG = laparoscopic sleeve gastrectomy, SAS = sleep apnea syndrome, T2DM = type 2 diabetes mellitus.

Descriptive statistics consisting of means, standard deviations, and percentages were calculated for variables. When the measurement data between the 2 groups obeyed a normal distribution and the variance was uniform, a paired *t* test or one-way ANOVA was used for comparisons. If they did not obey a normal distribution, the rank sum test was used, and the chi-square test or Fisher exact probability method was used for counting data. Statistical analyses were performed with SPSS (version 20; SPSS IBM). The LASSO regression model was used to screen out meaningful risk factors, and R software (version 3.6.3; https://www.R-project.org) was used to incorporate the selected risk factors into multivariate logistic regression analysis. *P* < .05 was the level of significance.

## 3 Results

The retrospective data showed that, compared with that before surgery, the serum VitB_12_ level of patients was significantly increased 1 month after surgery (*P* < .001); the serum VitB_12_ level of the patients improved 3 months after surgery; and there was no significant difference in serum VitB_12_ level 6 months after surgery (*P* = .945). The retrospective data are presented in Table 2.

**Table 2:** Retrospective data of serum vitamin B_12._

Follow-up	N (%)	Serum vitamin B_12_ (pg/mL)	*P* value
Preoperative	112 (100)	468.92 ± 122.22	<.001
1 mo		887.28 ± 390.55	
Preoperative	60 (53.57)	460.91 ± 123.98	.002
3 mo		579.49 ± 307.56	
Preoperative	40 (35.71)	482.79 ± 126.38	.945
6 mo		480.73 ± 178.01	

The results are expressed as mean ± SD or percentages. The level of significance is *P* < .05.

A comparison of the case data between the elevated serum VitB_12_ group and nonelevated serum VitB_12_ group revealed some interesting findings. There was no significant difference in demographic data between the 2 groups. More patients in the nonelevated serum VitB_12_ group had diabetes than in the elevated serum VitB_12_ group (*P* = .015). The postoperative serum folic acid level of the elevated serum VitB_12_ group was significantly higher than that of the nonelevated serum VitB_12_ group (*P* = .012). The postoperative serum VitB_12_ level of the elevated serum VitB_12_ group was significantly higher than that of the nonelevated serum VitB_12_ group (*P* < .001). The comparison of demographic and clinical data between the 2 groups is presented in Table 3.

**Table 3:** Comparison of demographic and clinical data between elevated serum vitamin B12 group and nonelevated serum vitamin B_12_ group.

	Preoperative Nonelevated group	Elevated group	*P* value	Postoperative 1 month Nonelevated group	Elevated group	*P* value
Gender (n)			.214			
Male	14	15				
Female	20	38				
Age (yr)	31.12 ± 8.93	31.40 ± 9.91	.895			
Height (cm)	167.71 ± 8.94	166.08 ± 8.41	.392			
Weight (kg)	108.08 ± 26.19	104.51 ± 23.43	.509	95.22 ± 23.85	91.91 ± 21.42	.503
BMI (kg/m^2^)	37.99 ± 6.42	37.60 ± 6.09	.775	33.47 ± 6.06	33.07 ± 5.68	.752
Mode of surgery (n)			.090			
LSG	17	32				
LSG + JJB	11	19				
LRYGB	6	2				
EWL%				32.81 ± 13.83	31.98 ± 9.40	.739
Hematological indexes						
Hemoglobin (g/L)	143.91 ± 15.18	140.81 ± 13.97	.332	144.65 ± 11.29	141.96 ± 11.28	.282
Folic acid (ng/mL)	7.86 ± 3.68	7.82 ± 3.59	.958	8.62 ± 4.41	11.25 ± 4.86	.012
Vitamin B_12_ (pg/mL)	450.47 ± 82.52	477.23 ± 64.29	.094	602.89 ± 99.03	1236.52 ± 349.62	<.001
ALT (U/L)	54.61 ± 38.52	50.51 ± 34.72	.607	46.50 ± 29.50	53.17 ± 35.03	.360
AST (U/L)	35.63 ± 20.68	30.34 ± 14.90	.169	35.10 ± 16.16	42.65 ± 23.90	.110
Creatinine (μmol/L)	59.04 ± 12.42	60.55 ± 13.99	.611	66.31 ± 17.6	91.94 ± 54.28	.558
Uric acid (μmol/L)	419.98 ± 110.05	433.38 ± 134.39	.628	479.59 ± 167.54	513.97 ± 202.26	.411
Triglyceride (mmol/L)	2.28 ± 1.31	1.86 ± 0.83	.07	1.36 ± 0.46	1.45 ± 0.56	.434
Cholesterol (mmol/L)	5.01 ± 0.97	4.90 ± 0.78	.577	4.07 ± 1.05	4.12 ± 0.81	.797
FBG (mmol/L)	7.10 ± 3.95	6.00 ± 2.21	.101	5.30 ± 0.85	5.06 ± 0.82	.204
HBA1c (%)	6.31 ± 1.71	5.95 ± 1.05	.359	5.75 ± 0.78	5.34 ± 0.9	.434
Preoperative complication (n)						
T2DM	12	7	.015			
HBp	7	12	.821			
Hyperuricemia	18	23	.384			
Hyperlipidemia	19	25	.428			
SAS	24	32	.332			
Fatty liver	33	46	.216			

The results are expressed as mean ± SD or percentages. The level of significance is *P* < .05. ALT = glutamic pyruvic transaminase, AST = glutamic oxaloacetic transaminase, BMI = body mass index, EWL = excess weight lost, FBG = fasting blood glucose, HBA1c = glycosylated hemoglobin, HBp = high blood pressure, JJB = jejunal bypass, LRYGB = laparoscopic Roux-en-Y gastric bypass, LSG = laparoscopic sleeve gastrectomy, SAS = sleep apnea syndrome, T2DM = type 2 diabetes mellitus.

When comparing the postoperative-related factors between the 2 groups, more patients had concurrent constipation in the elevated serum VitB_12_ group than in the nonelevated serum VitB_12_ group (χ^2^ = 14.027, *P* = .003). The comparison of postoperative-related factors between the 2 groups is presented in Table 4.

**Table 4:** The correlation between different postoperative factors and the increase of serum vitamin B_12._

	multivitamin tablets		Constipation		Reflux	
Group	Take	Not take	Yes	No	Yes	No
Control group	24	10	6	28	2	32
Case group	42	11	30	23	9	44
χ2 value	0.848		12.959		1.414	
*P* value	.357		<.001		.234	

The level of significance is *P* < .05. Constipation standard: Fewer than 3 defecations per week.^[15]^ Reflux standard: There are symptoms of acid regurgitation and heartburn after surgery and must take acid suppressants.

**Figure 1:**
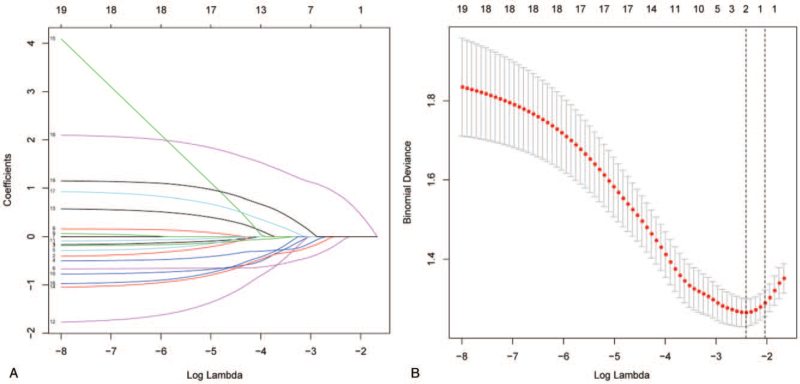
Demographic and clinical factor selection using the LASSO binary regression mode. (A) Optimal parameter (lambda) selection in the LASSO model used five-fold cross-validation via minimum criteria.^[16]^ The partial likelihood deviance (binomial deviance) curve was plotted versus log (lambda). Dotted vertical lines were drawn at the optimal values by using the minimum criteria and the 1 SE of the minimum criteria (the 1-SE criteria). (B) LASSO coefficient profiles of the 18 factors. A coefficient profile plot was produced against the log (lambda) sequence. Vertical line was drawn at the value selected using five-fold cross-validation, where optimal lambda resulted in 5 features with nonzero coefficients. LASSO = least absolute shrinkage and selection operator, SE = standard error.

Using the LASSO binary regression model to select related risk factors in the clinical data of patients, 18 factors were reduced to 2 potential risk factors with a nonzero coefficient in the LASSO regression model. The results of LASSO regression model analysis are presented in Figure 1 A and B.

**Figure 2:**
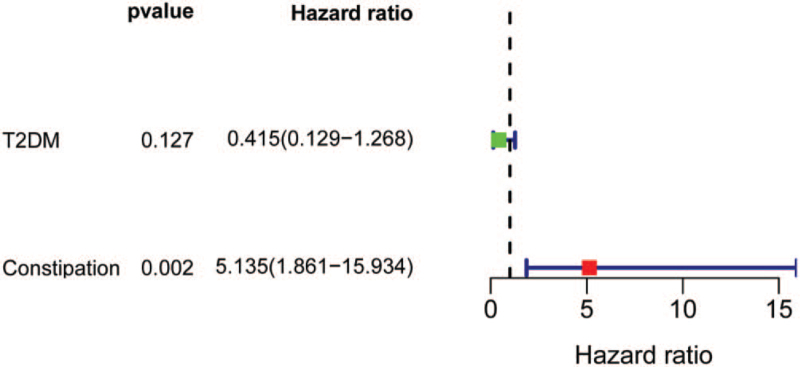
The results of multivariate logistic regression analysis.

These factors included preoperative diabetes mellitus and postoperative constipation. These 2 factors were incorporated into multivariate logistic regression analysis, and according to the analysis, postoperative concurrent constipation was an independent risk factor for the increase in serum VitB12 1 month after surgery (*P* = .002, hazard ratio = 5.135, 95% confidence interval: 1.861–15.934). The results of the multivariate logistic regression analysis are presented in Figure 2. Although postoperatively taking VitB12 was not a risk factor for postoperatively elevated VitB12. However, we suspect that taking VitB12 after surgery combined with constipation may lead to increased VitB12 after surgery. The difference between taking vitamins and constipation on the increase of VitB_12_ after surgery are presented in Supplemental Digital Content 2, http://links.lww.com/MD2/A788.

**Figure 3:**
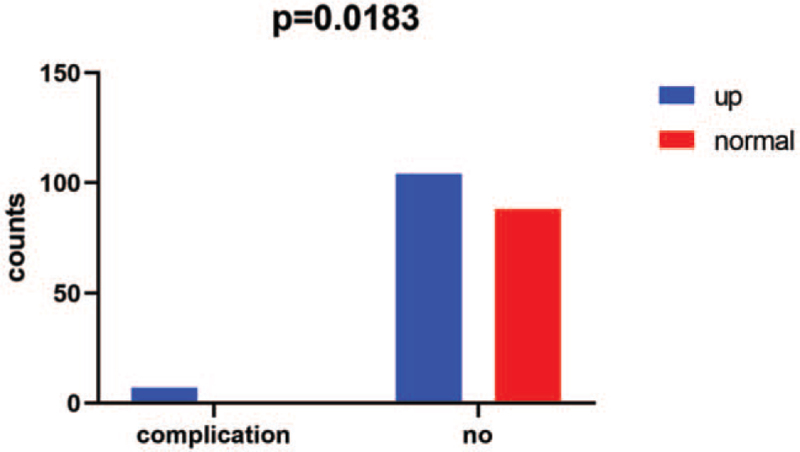
The patients with peripheral nerve symptoms after surgery.

Among the 199 patients in this study, 111 (55.8%) patients had higher-than-normal serum VitB_12_ levels 1 month after surgery. Among them, 7 patients had peripheral nerve symptoms, such as limb numbness, fatigue, pain, facial edema, and whole body stiffness. The 7 patients’ major clinical data are presented in Supplemental Digital Content 1, http://links.lww.com/MD2/A787. Their serum VitB_12_ levels were all >1500 pg/mL. None of the other 88 patients with normal serum VitB_12_ levels after surgery had the above symptoms. The patients with peripheral nerve symptoms after surgery are presented in Figure 3.

## 4 Discussion

VitB_12_, also referred to as cobalamin, is a water-soluble vitamin. VitB_12_ in nature is synthesized by microorganisms, but higher animals and plants cannot produce VitB_12_, which can only be supplemented by exogenous VitB_12_.^[17]^ The liver is the main place of VitB_12_ storage. VitB_12_ is mainly excreted through bile and reabsorbed in the ileum. This rarely lost hepatointestinal circulation allows VitB_12_ to be completely retained, so it takes many years, for even strict vegetarians, to develop VitB_12_ deficiency.^[18]^ Bariatric surgery removes about two-thirds of the stomach, mainly the fundus and body of the stomach. The decrease in gastric parietal cells and the secretion of gastric acid and internal factors after the operation coupled with the use of H2-receptor inhibitors and proton pump inhibitors can lead to postoperative malabsorption of VitB_12_.^[13]^ However, due to the slow consumption of VitB_12_ stored in the liver, VitB_12_ deficiency is usually not detected in the early stage after the operation (within 6 months after the operation).^[10]^ Similarly, none of the patients in our study had serum VitB_12_ deficiency 1 month after surgery. Since VitB_12_ is mainly absorbed in the duodenum and proximal jejunum, VitB_12_ deficiency is more common after laparoscopic RYGB than after other surgical methods.^[19]^ To prevent postoperative micronutrient deficiency, perioperative support guidelines for weight loss patients recommend taking 1 or 2 chewable multivitamin and mineral supplements every day.^[20]^ According to the recommendations of the guidelines, patients are required to consume a transparent liquid diet within 1 month after operation, supplemented with 60 g of protein powder and 2 multivitamin tablets daily.

There have been many studies on the phenomenon and mechanism of VitB_12_ deficiency after bariatric surgery. However, there are few reports on the phenomenon and causes of an abnormal increase in VitB_12_ after bariatric surgery. B vitamins are currently considered to be safe, and there are no tolerable upper levels that have been set, mainly due to the lack of systematic research on adverse reactions.^[21]^ In this study, we found that some patients undergoing bariatric surgery had increased serum VitB_12_ 1 month after surgery, which returned to a normal level within 3 to 6 months after surgery. During this period, some of the patients even had adverse reactions, such as limb numbness, fatigue, and tingling. Their serum VitB_12_ levels were all more than 1500 pg/mL, and through analysis, it was found that postoperative constipation was an independent risk factor for increased serum VitB_12_ within 1 month after bariatric surgery. Constipation is one of the common short-term complications after bariatric surgery.^[22]^ In this study, most of the patients suffered constipation within 1 month after surgery. It has been reported that patients with malabsorption surgery, such as biliopancreatic diversion and RYGB have a higher frequency of diarrhea and fecal incontinence than before the operation.^[23]^ Of the patients treated with B biliopancreatic diversion PD and RYGB, only 5% and 7% reported constipation after surgery, respectively.^[24]^ On the other hand, restrictive surgery, such as the gastric band or LSG, may make patients prone to constipation.^[25,26]^ In this case-control study, the majority of patients were treated with LSG or LSG + JJB.

LSG changes the shape of the stomach, reduces its volume, and alters its vagal innervation and quantity of secretory cells. These may increase intestinal transport.^[27]^ At the same time, increased postprandial levels of glucagon-like peptide-1 and peptide-YY after bariatric surgery can also delay intestinal transport.^[28]^ All these may lead to postoperative constipation. In addition, dietary fiber intake plays an important role in determining stool volume and intestinal transport time.^[29]^ Our patients require a clear and liquid diet within 1 month after bariatric surgery, and their dietary fiber intake is significantly reduced. It can also increase the incidence of postoperative constipation. What's more, it is reported that the symptoms of constipation, indigestion and vomiting in obese patients may be caused by thiamine deficiency.^[30]^ After RYGB and LSG treatment, 18% and 25.7% of the patients developed thiamine deficiency, respectively.^[31]^ Combined with the existing information, we postulate that since VitB_12_ is largely reabsorbed through the enterohepatic circulation, constipation leads to an increase in enterohepatic circulation, resulting in an increase in serum VitB_12_. In addition, 1 study pointed out that supplementation of B vitamins for 3 months after bariatric surgery can rapidly increase the level of serum VitB_12_.^[32]^ However, in this study, it was found that multivitamin supplementation within 1 month after surgery was not a risk factor for increased serum VitB_12_. Multivitamin supplementation may have a synergistic effect with constipation. Folic acid is a water-soluble B vitamin, which is mainly stored in the liver. Folic acid deficiency can lead to many side effects, but high folic acid levels have no obvious side effects. Allergic reactions and gastrointestinal symptoms, such as nausea and abdominal distension, are occasionally seen. Some researchers worry that excessive folic acid may mask the lack of VitB_12_.^[33]^ In this study, folic acid increased at the same time 1 month after surgery, but it did not exceed the normal level, which may be similar to the increase in VitB_12_.

Several strengths and limitations of this study should be noted. The same surgical team performed all the bariatric surgeries. The patient's postoperative management and follow-up data were also recorded by the same person. Limitations of the study include a small sample size and using single-center data, as well as having only short-term follow-up data and a lack of prospective studies to verify these etiological hypotheses.

## 5 Conclusion

In summary, the level of serum VitB_12_ after bariatric surgery is still an important clinical problem. Constipation is a risk factor for the increase in serum VitB_12_ in patients after this operation. To avoid postoperative vitamin-related complications, it is necessary to monitor the status of vitamins as soon as possible and solve problems such as constipation. High levels of VitB_12_ may cause peripheral nerve symptoms, such as limb numbness, fatigue, pain, facial edema, and whole body stiffness. Whether there are other toxic problems that require longer-term observation and study has not been elucidated. We should consider increasing the intake of dietary fiber to prevent and improve constipation after the operation and appropriately reducing VitB_12_ supplementation after the operation or formulating a multivitamin supplement program by stages after the operation to prevent excessive levels of VitB_12_. This topic is very important to improve the prognosis of patients after bariatric surgery.

## Acknowledgments

The authors would like to thank the efforts of the medical staff who followed the patients in The Third People's Hospital of Chengdu.

## Author contributions

**Data curation:** Jinhua Zhou, Rui Mao.

**Formal analysis:** Pengsen Guo, Huawu Yang, Jinhua Zhou, Dafang Zhan.

**Funding acquisition:** Pengsen Guo, Huawu Yang, Tongtong Zhang.

**Investigation:** Huawu Yang, Jinhua Zhou, Rui Mao, Dafang Zhan, Tongtong Zhang.

**Methodology:** Pengsen Guo, Rui Mao, Dafang Zhan.

**Resources:** Yanxi Ou.

**Validation:** Yanxi Ou, Yanjun Liu.

**Visualization:** Yanjun Liu.

**Writing – original draft:** Pengsen Guo, Jiang Yuan.

**Writing – review & editing:** Pengsen Guo.
